# A Case for Biopsy: Injectable Naltrexone-Induced Acute Eosinophilic Pneumonia

**DOI:** 10.7759/cureus.10221

**Published:** 2020-09-03

**Authors:** Pranay R Korpole, Souad Al-Bacha, Salim Hamadeh

**Affiliations:** 1 Internal Medicine, St. Mary Mercy Hospital, Livonia, USA; 2 Internal Medicine, Covenant Healthcare, Saginaw, USA; 3 Internal Medicine, Henry Ford Health System, Detroit, USA

**Keywords:** acute eosinophilic pneumonia, injectable naltrexone, vivitrol, bal, lung biopsy

## Abstract

Naltrexone is a semi-synthetic opioid that has competitive antagonist activity at mu opioid receptors. Naltrexone has proven to be efficacious in the treatment of alcohol and opioid dependence, and a long-acting injectable form of naltrexone was developed to overcome non-compliance. Therefore, injectable naltrexone has the potential to become an important medication for the treatment of opiate and alcohol dependence. Acute eosinophilic pneumonia (AEP) is a rare acute respiratory illness of varying severity that may lead to acute respiratory distress syndrome and death. Initially, AEP was thought to be idiopathic; however, it has become apparent that AEP can have identifiable causes including medications, infections, and other inhalational exposures, especially tobacco smoke. AEP is generally a diagnosis of exclusion confirmed by the presence of bronchoalveolar lavage (BAL) fluid eosinophilia. Recognition and elimination of the causative factor for AEP and providing glucocorticoid therapy are key principles in the management of AEP of non-infectious origin. Prognosis is generally excellent if AEP is diagnosed early and managed appropriately, even in patients with acute respiratory failure. The diagnosis of AEP is generally overlooked given the shared clinical attributes with acute lung injury due to other causes, including severe community-acquired pneumonia. A 32-year-old lady presented to the emergency department (ED) with symptoms of dyspnea, chest pain, cough, and subjective fevers since three days. She received a dose of intramuscular Naltrexone for the treatment of alcohol and opiate dependence on the day of symptom onset. Initially, she was noted to be hypoxic, and oxygen supplementation was initiated through a nasal cannula. While in the ED, she was placed on a non-rebreather mask because of worsening hypoxia. Chest imaging showed diffuse bilateral pulmonary infiltrates. Initial laboratory data were pertinent for elevated WBC count with mild peripheral eosinophilia. Antibiotics were administered for the treatment of suspected community-acquired pneumonia. Upon hospital admission, she was started on steroids for the management of suspected eosinophilic pneumonia secondary to injectable naltrexone. Bronchodilator therapy was initiated, and antibiotics were discontinued. The patient’s oxygen requirements improved. Pulmonology consultation was requested, and the patient underwent bronchoscopy. BAL studies showed predominance of lymphocytes with no eosinophils. However, lung biopsy showed findings consistent with drug-induced eosinophilic pneumonitis. The patient’s hypoxia resolved with steroid therapy. The patient was discharged with a course of oral steroids, albuterol inhaler, and outpatient pulmonology follow-up.

## Introduction

Opioid dependence is a significant cause of morbidity and mortality. According to the Global Burden of Diseases, Injuries, and Risk Factors Study, in 2017, approximately 40·5 million people were opioid-dependent and 109,500 people died from opioid overdose [1]. Naltrexone is a semi-synthetic opioid that has competitive antagonist activity at mu opioid receptors [2]. Studies have demonstrated the efficacy of naltrexone in the treatment of alcohol and opioid dependence [2]. A long-acting injectable form of naltrexone with activity for 30 days was developed to overcome the issue of non-compliance [2]. Therefore, injectable naltrexone has the potential to become an important medication for the treatment of opiate and alcohol dependence [2].

Acute eosinophilic pneumonia (AEP) is a rare acute respiratory illness of varying severity that may lead to acute respiratory distress syndrome and death [3]. AEP was initially described as a discrete clinical entity in 1989 associated with acute febrile illness, diffuse pulmonary infiltrates, and acute respiratory failure characterized by bronchoalveolar lavage (BAL) fluid eosinophilia and prompt clinical improvement after corticosteroid therapy [3]. The initial four cases of AEP were thought to be idiopathic; however, it has become apparent that AEP can have identifiable causes including medications, infections, and other inhalational exposures, especially tobacco smoke [3]. AEP pathogenesis is not well understood but probably involves different pathways depending on the underlying cause [3]. Eosinophil recruitment to the lung is initiated secondary to airway epithelial injury, endothelial injury, and release of IL-33 (interleukin 33). The recruited eosinophils subsequently degranulate, leading to inflammation of the lung tissue and clinical features of the disease [3]. AEP is generally a diagnosis of exclusion confirmed by the presence of BAL fluid eosinophilia [3] Peripheral blood eosinophilia may suggest the diagnosis of AEP; however, it may not always be present, especially in smoking-related AEP [3]. Recognition and elimination of the causative factor for AEP and providing glucocorticoid therapy are key principles in the management of AEP of non-infectious origin [3]. Prognosis is generally excellent if AEP is diagnosed early and managed appropriately, even in patients with acute respiratory failure [3].

AEP secondary to injectable naltrexone use is rare. The diagnosis of AEP is generally overlooked given the shared clinical attributes with acute lung injury due to other causes, including severe community-acquired pneumonia [3].

## Case presentation

**Figure 1:**
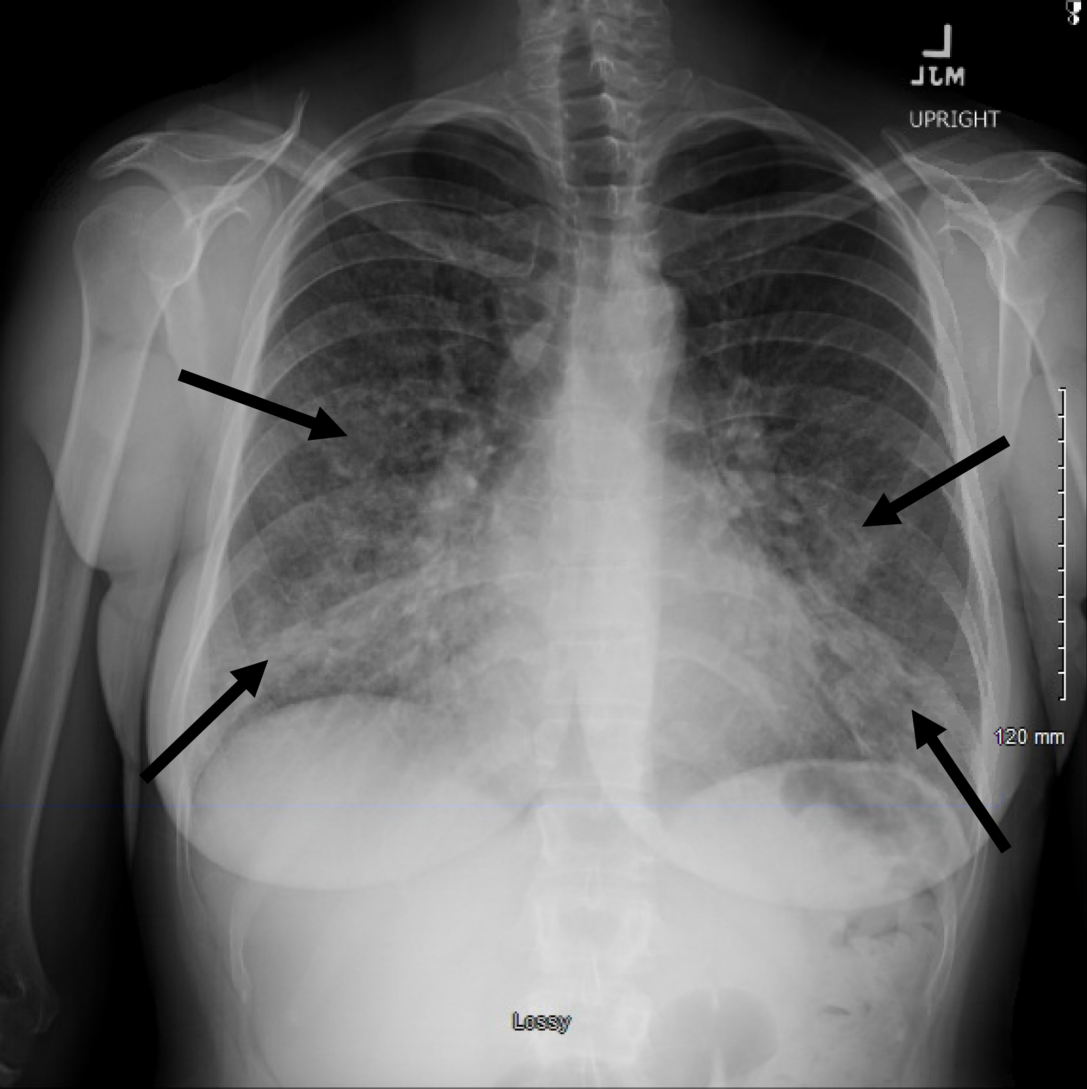
Chest X-ray (PA view) demonstrating diffuse bilateral lung infiltrates (indicated by arrows). PA, posteroanterior

**Figure 2:**
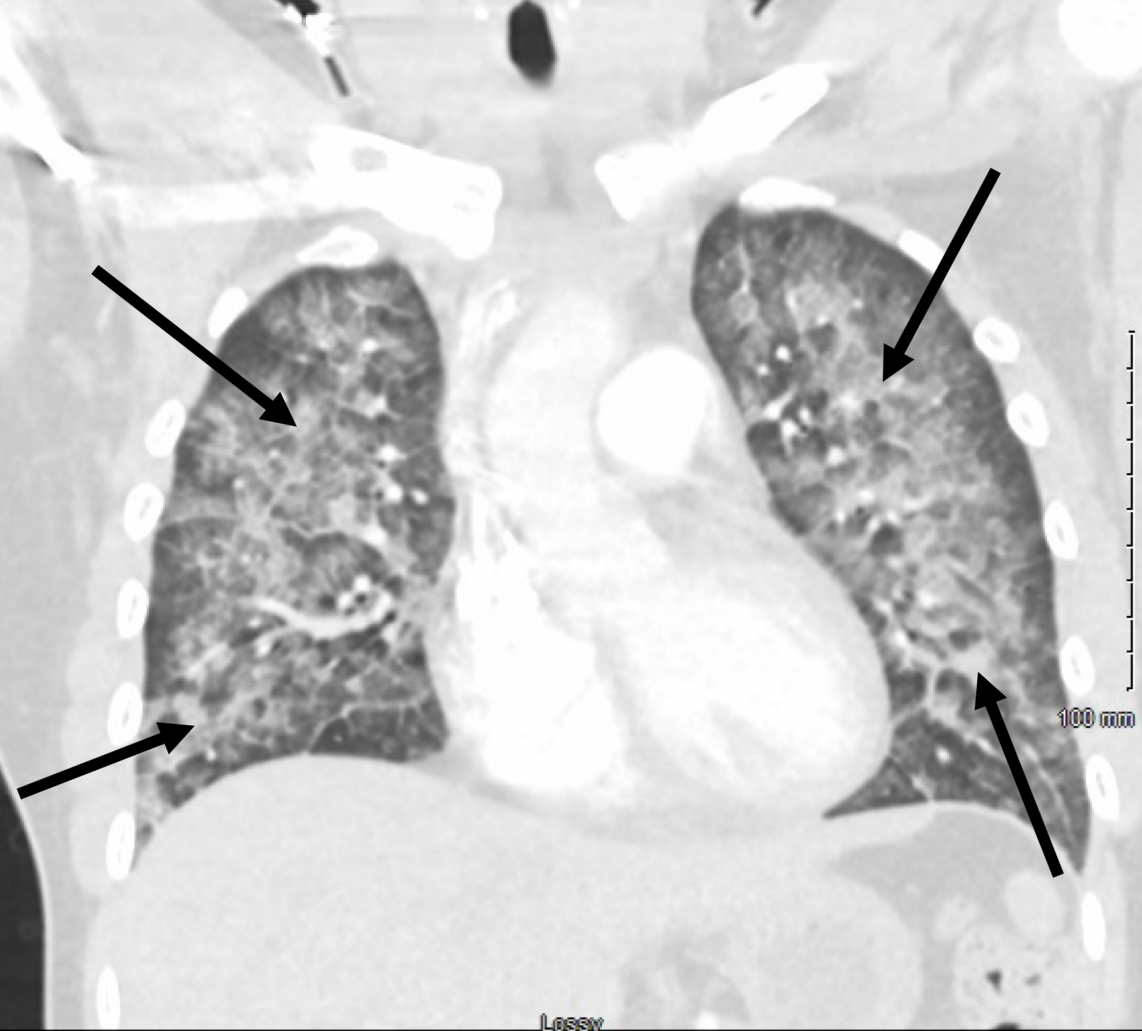
Chest CT (coronal plane image) showing diffuse bilateral pulmonary infiltrates (indicated by arrows).

**Figure 3:**
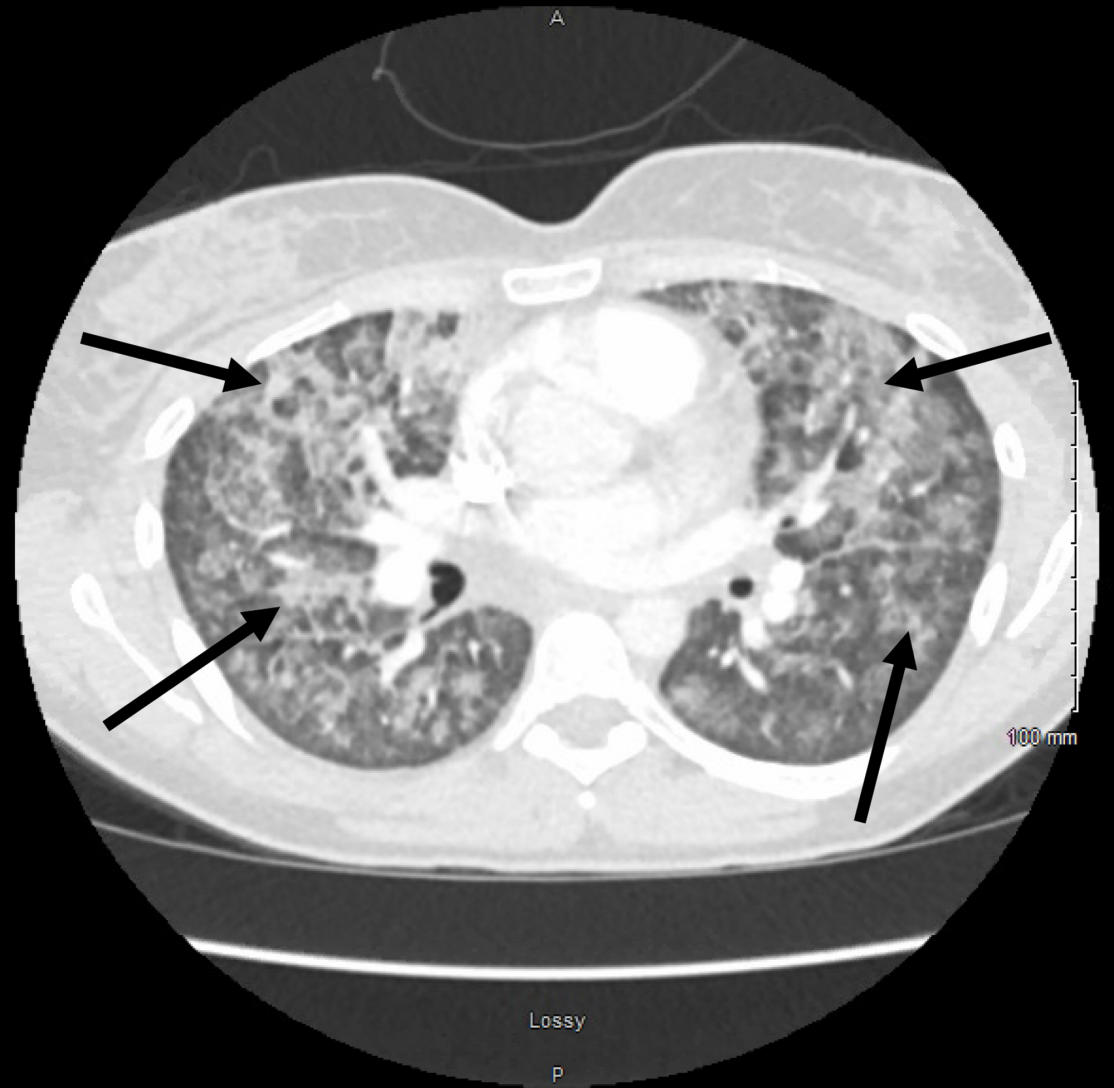
Chest CT (transverse plane image) showing diffuse bilateral pulmonary infiltrates (indicated by arrows).

A 32-year-old lady presented to the emergency department (ED) with symptoms of dyspnea, chest pain, cough, and subjective fevers since three days. The patient was reportedly in good health before she received a dose of intramuscular naltrexone for the treatment of alcohol and opiate dependence on the day of symptom onset. The patient had been smoking cigarettes consistently for the past six years. On arrival, she was noted to be hypoxic, and oxygen supplementation was initiated through a nasal cannula. Examination was pertinent for tachypnea and bilateral crackles. The patient was afebrile. While in the ED, she was placed on a non-rebreather mask because of worsening hypoxia. Chest X-ray showed diffuse bilateral pulmonary infiltrates (Figure 1). A CT angiogram of the chest was ordered, which was negative for pulmonary embolism but showed diffuse bilateral pulmonary infiltrates as well (Figures 2, 3). Initial laboratory data were pertinent for elevated WBC count with mild peripheral eosinophilia (700 cells/microliter). Levofloxacin was administered intravenously for the treatment of suspected community-acquired pneumonia.

**Figure 4:**
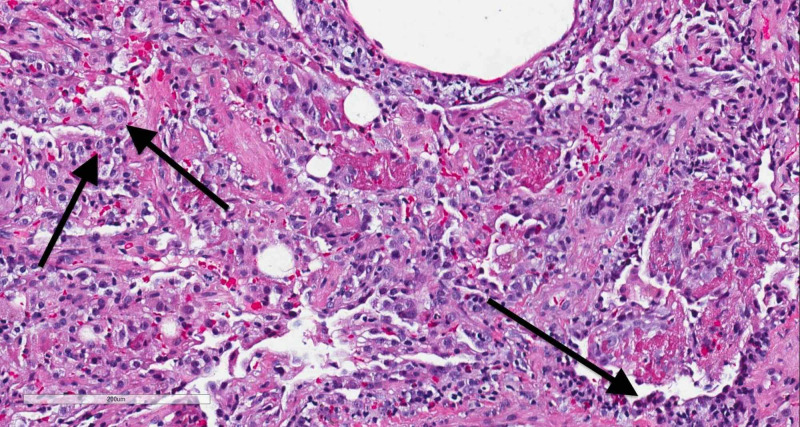
Histology of lung biopsy sample showing the presence of eosinophils (indicated by arrows) in the lung parenchyma.

The patient was admitted to the medicine service and was started on intravenous methylprednisolone for the management of suspected AEP secondary to injectable naltrexone given the temporal relationship between administration of the medication and onset of symptoms. Bronchodilator therapy was initiated for wheezing. Antibiotics were discontinued upon admission. The patient’s oxygen requirements improved. Pulmonology consultation was requested, and the patient underwent bronchoscopy. BAL studies showed predominance of lymphocytes (62%) followed by neutrophils (32%) with no eosinophils, which was not suggestive of AEP. However, histology of the lung biopsy sample showed the presence of eosinophils in the lung parenchyma consistent with drug-induced eosinophilic pneumonitis (Figure 4). HIV viral load testing and hepatitis C serology were negative, BAL bacterial cultures (including tuberculosis) remained sterile, and the BAL fungal culture grew rare yeast, which were considered to be contaminants. BAL fluid testing for *Pneumocystis jirovecii* was negative as well. The patient’s hypoxia resolved with steroid therapy. The patient was discharged with a course of oral prednisone, albuterol inhaler, and outpatient pulmonology follow-up.

## Discussion

The modified Philit criteria are currently used to diagnose “definite” AEP and are as follows : (1) acute respiratory illness of less than or equal to one month of duration, (2) pulmonary infiltrates on chest radiography or CT, (3) pulmonary eosinophilia as demonstrated by more than 25% eosinophils in BAL fluid (can be accompanied by variably increased percentages of lymphocytes and neutrophils) or eosinophilic pneumonia on lung biopsy (bronchoscopic or surgical), and (4) absence of other specific pulmonary eosinophilic diseases including eosinophilic granulomatosis with polyangiitis (Churg-Strauss syndrome), hypereosinophilic syndrome, and allergic bronchopulmonary aspergillosis [3].

Drug-induced AEP is diagnosed using the Solomon and Schwarz criteria, which are as follows: (1) presence of simple, acute, or chronic eosinophilic pneumonia by diagnostic criteria, (2) presence of a potential candidate drug or toxin in an appropriate time frame, (3) exclusion of other causes of eosinophilic pneumonia, such as fungal or parasitic pneumonia, (4) clinical improvement after cessation of the drug or toxin, and (5) recurrence with re-challenge to the drug or toxin [3]. However, in clinical practice, clinical improvement after cessation of exposure to the suspected agent is usually sufficient in the diagnosis without the need for re-challenge [3].

Four cases of eosinophilic pneumonia have been reported with the use of injectable naltrexone [4-7]. Three of these cases have been described as case reports in the medical literature [5-7]. The patient’s presentation in Kim et al.’s case report was consistent with chronic eosinophilic pneumonia, whereas the patient’s presentation in Horsley and Wesselius’s and Esposito et al.’s case reports were in keeping with AEP [5-7]. Our patient met all the criteria included in the modified Philit criteria for the diagnosis of “definite” AEP [3]. All but one of criteria proposed by Solomon and Schwarz for the diagnosis drug-induced AEP were also fulfilled in our case - the only criterion excluded was the one involving recurrence of clinical syndrome with re-exposure to the offending agent, which was considered to be impractical [3].

Pulmonary eosinophilia as demonstrated by eosinophil count comprising greater than 25% of BAL fluid leukocytes is a common finding in AEP, which obviates the need for a lung biopsy [3]. Pulmonary eosinophilia as demonstrated by BAL fluid analysis was notably absent in our patient but clinched the diagnosis in the other three case reports highlighted above [5-7]. We suspect that the absence of BAL fluid eosinophils in our case was secondary to the administration of steroids soon after hospital admission, a little more than 48 hours before bronchoscopy was pursued. Our case demonstrates the importance of pursuing a lung biopsy when there is a high suspicion for AEP despite non-diagnostic BAL fluid findings.

## Conclusions

Injectable naltrexone has the potential to become an important medication in the management of alcohol and opioid dependence. The use of injectable naltrexone is associated with the potentially fatal side effect of AEP. The diagnosis of AEP may be overlooked given its shared clinical attributes with other causes of acute lung injury including community-acquired pneumonia. Patients with AEP generally have a favorable prognosis if the disease is diagnosed early in the clinical course. In our patient, AEP was suspected at the time of hospital admission, which led to early initiation of treatment with intravenous steroids. Our patient's respiratory symptoms and oxygen requirements rapidly improved after the initiation of intravenous steroid therapy.

The diagnosis of AEP is generally established by the demonstration of eosinophilia in the BAL fluid analysis. However, in our case, BAL fluid eosinophilia was conspicuously absent, and the diagnosis of AEP was eventually confirmed by the results of the lung biopsy. We suspect that the absence of BAL fluid eosinophilia in our case is possibly secondary to the initiation of empiric intravenous steroids early in the clinical course of the disease prior to bronchoscopy. Lung biopsy should be pursued in cases where high suspicion of AEP persists despite the absence of BAL fluid eosinophilia.
